# Effect of the Sequential Inoculation of Non-*Saccharomyces/Saccharomyces* on the Anthocyans and Stilbenes Composition of Tempranillo Wines

**DOI:** 10.3389/fmicb.2019.00773

**Published:** 2019-04-09

**Authors:** Rocío Escribano-Viana, Javier Portu, Patrocinio Garijo, Rosa López, Pilar Santamaría, Isabel López-Alfaro, Ana Rosa Gutiérrez, Lucía González-Arenzana

**Affiliations:** Instituto de Ciencias de la Vid y el Vino, CSIC, Gobierno de La Rioja, Universidad de La Rioja, Logroño, Spain

**Keywords:** wine color, anthocyans, stilbenes, *Zygosaccharomyces bailii*, *Metschnikowia pulcherrima*, *Torulaspora delbrueckii*, *Candida zeylanoides*

## Abstract

The phenolic compounds of red wines are responsible for their color, astringency, and antioxidant properties. The fermentative yeasts might be used to modulate wines in terms of their color, aroma and probably healthy properties. In this study, six non-*Saccharomyces* species were tested because they might enhance the properties of red Tempranillo wines from Rioja. The results confirmed that the anthocyanins and stilbenes composition of wine can be modulated with the use of a specific fermentation starter. *Metschnikowia pulcherrima*, *Zygosaccharomyces bailii*, *Candida zeylanoides*, and *Torulaspora delbrueckii* achieved the greatest improvements of the monomeric anthocyanin composition, and the latter three yeast species achieved the best results of stilbene composition when compared to *S. cerevisiae* and the other non-*Saccharomyces* yeasts. Overall, results suggested that the use of *M. pulcherrima, Z. bailii, C. zeylanoides* and *T. delbrueckii* as fermentation starters could be of great interest to achieve wines with better color and likely healthy properties.

## Introduction

The phenolic compounds of red wines are responsible for their color, bitterness, astringency, antioxidant properties and aging behavior (Gonzalo-Diago et al., 2017). Wine color is one of the most relevant property that influences first consumer valoration (Kennedy, 2010). In addition, red wines with high phenolic content are more attractive due to their role in reducing the risk of cardiovascular disease and cancer (Dell’Agli et al., 2004).

Winemaking techniques influence the phenolic content of red wines, thereby determining wine quality. The quantity of phenolic compounds that are transferred from grape to wine during maceration varies according to the conditions of the process, and according to the microbial population present in the wine. Caridi et al. (2017) validated the main role that wine yeasts play in enhancing the quality of red wine from low-pigmented grapes. Thus, in recent years, yeast selection has included the development of techniques for detecting strains that might improve wines in terms of their color, structure, aroma and probable health properties (Suárez-Lepe and Morata, 2012).

Yeasts have an influence on wine’s final chromatic characteristics through different routes. Some cases in point are the pectinase enzymes that they release during maceration (Belda et al., 2016). The role that yeast cell walls play in anthocyanin and tannins adsorption is due to the chemical interaction between yeast and polyphenols and to the metabolites that yeasts produce during alcoholic fermentation (AF) (Morata et al., 2003). For instance, some non-*Saccharomyces* (*S.*) such as *Lachancea (L.) thermotolerans* and *Torulaspora (T.) delbrueckii* yeasts enhanced the phenolic compositions of red Rioja wines (Garofalo et al., 2016). In previous research, it was also observed that the sequential inoculation of these same species, triggered different aromatic profiles than fermentation trials only with *S. cerevisiae* (Escribano-Viana et al., 2018).

Red wine color depends on anthocyanin extraction from grape skin and its stabilization in wine in a colored form. Pectinase enzymes have shown a considerable influence on releasing compounds entrapped in grape skin, facilitating the liberation of phenolic compounds. These enzymes come from grapes, commercial preparations and also from microorganisms (Belda et al., 2016). On the other hand, retention of pigments in yeast cell walls represents a direct loss of color (Morata et al., 2016). The negative charges of yeast cell walls provide the strains its adsorption ability toward the positively charged polyphenols of wines. Caridi et al. (2015) found significant differences between strains in terms of their aptitude to change wine color.

In addition, some metabolites, such as pyruvic acid and acetaldehyde, produced by yeasts during the glycolytic stage of AF, may condense with grape anthocyanins to produce highly stable pyranoanthocyanin adducts, such as vitisin A and B respectively (Morata et al., 2007). This reaction can lead to the stabilization of anthocyanins during wine aging. On account of this, the yeast ability to synthesize carbonyl compounds during fermentation directly impacts the formation of stable pigments, as they act as precursors of pyranoanthocyanins (Morata et al., 2016). Stabilization of anthocyanins can occur through reaction between anthocyanins and tannins to form pigmented tannins and through copigmentation of anthocyanins (Kennedy, 2010). Carew et al. (2013) showed that the yeast strain significantly affects both the concentration and composition of wine tannins as well as the degree of tannin polymerization. Moreover, vinylphenolic pyroanthocyanins adducts are condensation products between vinylphenols and anthocyanins that show great color stability (Morata et al., 2007). Vinylphenols are originated during fermentation by the action of the hydroxycinnamate decarboxylase enzyme (HCDC) of yeasts. Yeasts with HCDC activity can also be used to decarboxylate hydroxycinnamic acids and form vinylphenols that condense with grape anthocyanins to produce vinylphenolic pyroanthocyanin adducts.

Resveratrol is a stilbene compound cataloged as a polyphenol which has shown its health benefits *in vitro* and *in vivo* studies (Poulsen et al., 2014). Stilbenes are phytoalexins that are associated with plant resistance to fungal diseases as well as response to abiotic stresses. In grape berries, stilbene derivatives are mainly accumulated in the skin according to the localization of stilbene synthase, although they have also been identified in seeds. The main stilbenes are both *trans* and *cis* forms of resveratrol and of its glucoside piceid. In wine, stilbenes concentration is greatly determined by the initial grape stilbene amount, but also by the winemaking process and fermentative microorganisms. In this respect, Sun et al. (2003) found that wines made with skin fermentation had greater amount of resveratrol than those made by carbonic maceration technique. Moreover, yeasts endowed with β-glucosidase activity enhance free resveratrol concentration in wine (Gaensly et al., 2015) and can reduce the risk of some diseases and modify the antioxidant capacity of wine (Brandolini et al., 2007).

Nowadays, the selection of new inocula should be carried out for new characteristics such as the potential to improve wine color and polyphenol content while enhancing quality and healthiness (Caridi and Sidari, 2009). Aimed to avoid wine homogenization, non-*Saccharomyces* culture starters with different biotechnological characteristics are being selected by oenologists and researchers although they should be combined with conventional fermentative *Saccharomyces (S.) cerevisiae* to avoid sluggish and spoilt fermentations (Rossouw and Bauer, 2016). The use of starters of selected non-*Saccharomyces* combined with *S. cerevisiae* has been shown as a way to produce high quality wines (Canonico et al., 2016). So that in the current research, six previously selected autochthonous non-*Saccharomyces* yeast species (González-Arenzana et al., 2016; Escribano et al., 2017) have been sequentially and individually inoculated and also one mixed-inoculum has been tested. The non-*Saccharomyces* yeasts were inoculated at the beginning of fermentation and 3 days later the inoculation of *S. cerevisiae* was conducted.

## Materials and Methods

### Fermentation Trials

Ten individual fermentation trials were done in duplicate (*n* = 2) in small fermenters (Sampaio et al., 2007). Grapes from red Tempranillo variety were harvested in their optimum maturity moment. Then, in the experimental winery of the ICVV, those grapes were crushed and destemmed and liquid must was separated from solid grape skins (without pressing). After this, 2 l of must and 500 g of grape skins were measured and introduced into each flask. This initial grape must had 24.5 Brix or 14.5% probable alcoholic strength, 2.26 g/L malic acid, 4.9 total acidity expressed as g/L of tartaric acid, 3.73 pH and 181 mg/L of assimilable yeast nitrogen (including amino acids, except proline, and ammonia). Temperature was set at 25°C.

Eleven different assays were performed as it was described by Escribano-Viana et al. (2018). with ten oenological strains belonging to the following seven different yeast species: *Metschnikowia* (*M*.) *pulcherrima, T.* *delbrueckii*, *L. thermotolerans*, *Zygosaccharomyces* (*Z.*) *bailii*, *Williopsis* (*W*.) *pratensis*, *Candida* (*C*.) *zeylanoides*, and *S*. *cerevisiae* (VRB, Lallemand Bio S.L.). Two strains of every yeast species, one of *S. cerevisiae* and a combination of *L. thermotolerans* and *T. delbrueckii* combined in percentages 30 and 70% were tested. Every strain (except SVRB) were selected to enhance and diversify the quality of wines in the Rioja “Qualified” Designation of Origin (D.O. Ca.) Rioja, Spain and they were storage in the ICVV (Instituto de Ciencias de la Vid y el Vino) collection.

The pre-culture of non-*Saccharomyces* yeasts was carried out in YPD liquid medium at 25°C for 48 h before flask inoculation with 10^6^ cells/ml. Nine pure cultures and one mixed culture of *L. thermotolerans* and *T. delbrueckii* (3 × 10^5^ and 7 × 10^5^ cells/mL, respectively, 30/70) were tested. In other assay, *S. cerevisiae* VRB commercial yeast was initially inoculated following the manufacturer’s instructions. Three days after the first inoculation, the commercial *S. cerevisiae* was inoculated in a proportion of 1 × 10^6^ cells/mL. During AF, the Brix degree decreases because of the conversion of sugars into ethanol. This parameter was daily analyzed with a digital refractometer. The reduction in the Brix degree was used for representing the fermentation kinetic. 14 days after inoculation wines were pressed, sulphited at 30 mg/L and left to settle for a week. Then, the wines were bottled (0.75 L) and chemically analyzed.

### Sampling

Samples of 50 mL of the 11 assays were taken under aseptic conditions at three different moments: after filling flasks with must and skins and adding the inoculated yeast (day 1), before inoculating commercial *S. cerevisiae* (day 3) and 5 days after the beginning of the study (day 5).

Serial dilutions of samples were spreaded onto plates of a chloramphenicol glucose agar medium that were incubated at 25°C for 48 h. Colony forming units (CFU) were then counted. Precisely from every plate containing between 10 and 100 yeast CFU, 10 colonies were randomly selected (20 colonies for the combination L&T). The quick DNA extraction from the fresh yeast culture was conducted as López et al. (2008) suggested. Region D1/D2 of the 26S rDNA gene was amplified by PCR using primers and conditions described by Kurtzman et al. (2010). Then, the PCR products were purified and sequenced by Macrogen Inc. facilities (Amsterdam, Netherlands). The BLAST tool of the Gen Bank platform was employed (Altschul et al., 1990) to determine the species identification it was considered adequate when gene sequences showed identities of at least 98% with some of the sequences included in the Gen Bank database. To determine identities as correct, phylogenetic maximum likelihood tree was built with results of Gen Bank to corroborate the evolutionary correspondence.

### Oenological Parameters of Wines

Wines were characterized by the alcohol strength by volume (ABV), pH, total acidity, color intensity (CI) and hue following Council Regulation (EC) N° 479/2008 (McLaren and Rigg, 1976; Commission Regulation, 2009). The acetic, L (-) malic acid and D(-) and L(+) lactic acids were measured by an enzymatic method with an automated clinical chemistry analyser (Miura One, TDI, Spain) and tartaric acid by the Rebelein method (Lipka and Tanner, 1974). Total phenolics were determined as total polyphenol index (TPI) by spectrophotometric absorbance at 280 nm after dilution of samples.

### Analysis of Monomeric Anthocyanins and Stilbenes in Wine by HPLC-DAD

Anthocyanins were analyzed using an Agilent 1260 Infinity chromatograph, equipped with a diode array detector (DAD). The chromatographic procedure was as described by Portu et al. (2016) using a Licrospher^®^100 RP-18 reversed-phase column (250 × 4.0 mm; 5 μm packing; Agilent) with pre-column Licrospher^®^100 RP-18 (4 × 4 mm; 5 μm packing; Agilent). Column temperature was set at 40°C, the flow rate was established at 0.630 mL/min and the injection volume was 10 μL. Eluents used were (A) acetonitrile/water/formic acid (3:88.5:8.5, v/v/v), and (B) acetonitrile/water/formic acid (50:41.5:8.5, v/v/v). The linear solvent gradient was as follows: 0 min, 6% B; 15 min, 30% B; 30 min, 50% B; 35 min, 60% B; 38 min, 60% B; 46 min, 6% B.

Regarding stilbene analysis, a previous purification step was conducted by solid phase extraction (SPE) with PCX SPE cartridges (500 mg, 6 mL; Bond Elut Plexa, Agilent, Palo Alto, CA, United States) placed in a Gilson GX-271 Spec (Gilson Inc, Middleton, WI, United States) (Portu et al., 2015). The fraction obtained from the SPE step was analyzed by HPLC-DAD using an Agilent 1260 Infinity. The methodology was adapted from Portu et al. (2017). Wine samples were injected into a Licrospher^®^100 RP-18 reversed-phase column (Agilent) with pre-column Licrospher^®^100 RP-18 (Agilent), both thermostated at 40°C. Flow rate was at 0.500 mL/min and injection volume was 20 μL. Water/acetonitrile/formic acid (100:10:0.1, v/v/v) was the solvent A and acetonitrile was the solvent B. The linear solvent gradient was as follows: 0 min, 0% B; 20.8 min, 16% B; 32.8 min, 16% B; 49.4 min, 42% B; 60 min, 0% B.

Anthocyanins and stilbenes were identified according to the retention times of the available pure compounds and the UV–Vis data obtained from authentic standards and/or published in previous studies of β-glucosidase activity. Anthocyanins were quantified at 520 nm as malvidin-3-*O*-glucoside (Extrasynthèse, Genay, France); *cis*-piceid and *cis*-resveratrol were quantified at 305 nm as their corresponding *trans* isomers. Concentrations were expressed as milligrams per liter of wine (mg/L). The results correspond to the average of the analyses of two samples (*n* = 2).

### Statistical Analysis

Analysis of variance (ANOVA) was carried out for all the parameters analyzed for the different assays with the IBM^®^SPSS^®^Statistic version 23. Significant differences were established by using the Tukey *post hoc* test (*p* < 0.01).

## Results

### Implantation of Inoculated Yeasts and Strains

Data of implantation and kinetics control are included in a previous published research that was aimed at the study of the aroma evolution through AF (Escribano-Viana et al., 2018).

The first sampling showed that each inoculated species was implanted with different percentages. For instance, *T. delbrueckii* and *L. thermotolerans* reached total implantation (100%); *S. cerevisiae* was 90% of the yeast population; *C. zeylanoides* reached 85%; *M. pulcherrima* 70%, *W. pratensis* 60%, and *Z.* *bailii* 35%. At this first sampling stage, the mixed inoculum seeded in a 30/70 ratio maintained this ratio.

At the second sampling moment, *S. cerevisiae* was 100 % of the yeast population, *C. zeylanoides* was 90%, *M. pulcherrima*, *T. delbrueckii*, and *L. thermotolerans* was around 80%, *Z. bailii* reached 30%, *W. pratensis* 10% and the mixed inoculum maintained the initial ratio of 30/70.

Finally, at the third sampling moment, *S. cerevisiae* was 100% of the yeast population, *T. delbrueckii* 40% and *L. thermotolerans* 30%. The mixed inoculum LT was in a ratio of 30/55 and the remaining 4 yeast species were not found in the samples. The implantation of the two genotypes of each species were significantly equal.

### Oenological Parameters of Wines

The wine initially vinified with *M. pulcherrima* had the lowest glycerine and reducing sugars content but the highest TPI value (Table 1). The sequential vinification with *T. delbrueckii* provided wines with the highest pH and glycerine concentration. In the case of *L. thermotolerans*, the wine had the lowest pH but in contrast, the reducing sugars, the total acidity, the lactic and acetic acids and the acetaldehyde content were the highest of the study. The mixed L&T culture provided an intermediate content of lactic acid and glycerine compared to the individual culture inoculation. The resulting wines made with the *Z. bailii* and *W. pratensis* sequential inoculation had the highest ethanol content. Eventually, the wine inoculated initially and sequentially with *S. cerevisiae* showed the highest ethanol and malic acid content and the lowest reducing sugar, acetaldehyde, and TPI values.

**Table 1:** Average oenological parameters assessed for wines sequentially tested with non-*Saccharomyces* species (*M.p*, *Metschnikowia pulcherrima;* *T.d*, *Torulaspora delbrueckii;* *L.th, Lachancea thermotolerans;* *L&T*, *L.th/T.d* 30/70; *Z.b*, *Zygosaccharomyces bailii;* *W.p*, *Williopsis pratensis;* *C.z*, *Candida zeylanoides*) and *S.c, Saccharomyces cerevisiae* with their standard deviation and results of statistical analysis.

Oenological parameters	*M.p*	*T.d*	*L.th*	*L&T*	*Z.b*	*W.p*	*C.z*	*S.c*
Ethanol content (v/v)	14.1 ± 0.0a	14.1 ± 0.1a	14.1 ± 0.1a	14.2 ± 0.0ab	14.3 ± 0.0b	14.3 ± 0.0b	14.3 ± 0.0b	14.3 ± 0.0b
Reducing sugars (mg/L)	1.78 ± 0.05a	2.08 ± 0.21a	3.68 ± 0.67b	2.58 ± 0.67ab	2.00 ± 0.00a	2.05 ± 0.21a	1.80 ± 0.14a	1.80 ± 0.00a
pH	3.69 ± 0.01bcd	3.75 ± 0.01e	3.46 ± 0.04a	3.63 ± 0.01b	3.70 ± 0.01de	3.67 ± 0.03bc	3.69 ± 0.00bcd	3.72 ± 0.01de
Total acidity (g/L tartaric acid)	5.26 ± 0.37ab	5.36 ± 0.09ab	6.90 ± 0.26c	5.71 ± 0.22b	5.32 ± 0.27ab	4.98 ± 0.08a	5.06 ± 0.05a	5.23 ± 0.21ab
Acetic acid (mg/L)	0.11 ± 0.02ab	0.09 ± 0.01a	0.19 ± 0.04b	0.13 ± 0.02ab	0.12 ± 0.04ab	0.14 ± 0.01ab	0.11 ± 0.07ab	0.16 ± 0.01ab
Malic acid (g/L)	1.87 ± 0.13bc	2.15 ± 0.04ab	1.88 ± 0.19ab	2.06 ± 0.15bc	1.62 ± 0.02a	1.46 ± 0.06a	1.44 ± 0.01a	2.46 ± 0.07c
Lactic acid (g/L)	0.17 ± 0.15a	0.20 ± 0.05a	3.38 ± 0.78c	1.11 ± 0.06b	0.16 ± 0.08a	0.04 ± 0.11a	0.17 ± 0.04a	0.16 ± 0.00a
Acetaldehyde (g/L)	12.2 ± 2.2b	23.8 ± 1.5a	29.5 ± 8.1b	24.8 ± 4.6ab	23.0 ± 1.4ab	14.0 ± 2.8ab	14.5 ± 2.1ab	11.0 ± 1.41a
Glycerine (g/L)	6.45 ± 3.63a	8.75 ± 0.24d	7.73 ± 0.13bc	8.2 ± 0.19cd	7.80 ± 0.14bc	8.00 ± 0.14c	7.85 ± 0.36bc	7.55 ± 0.07b
Hue	0.63 ± 0.15	0.61 ± 0.01	0.56 ± 0.05	0.56 ± 0.14	0.64 ± 0.01	0.74 ± 0.04	0.67 ± 0.01	0.71 ± 0.00
TPI	43.5 ± 1.3b	40 ± 0b	34.3 ± 1.9a	33.9 ± 1.8a	41.2 ± 0b	33.7 ± 0.6a	41.9 ± 3.1b	33.1 ± 1.18a
CI	7.98 ± 0.62	7.70 ± 0.34	7.53 ± 1.25	6.43 ± 0.81	7.40 ± 0.00	5.30 ± 0.42	7.25 ± 0.50	5.30 ± 0.28


*Different letters mean significant differences between the samples (*p* < 0.01). No letters mean no significant differences.*

### Anthocyan and Stilbene Content After Alcoholic Fermentation

**FIGURE 1:**
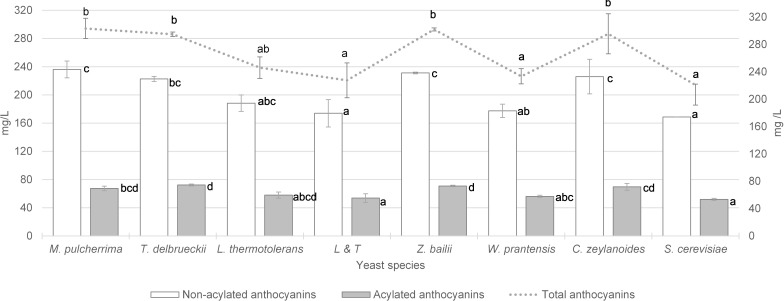
Total anthocyanins content (mg/L) and content of non-acetylated and acetylated anthocyanins in wine vinified with different yeasts species. Error bars represent the standard deviation (*n* = 2). Different letters mean significant differences between the samples (*p* ≤ 0.01). No letters mean no significant differences.

The total anthocyan content, for both the non-acylated and acylated anthocyans are displayed in Figure 1. The vinification performed with *S. cerevisiae* yeast achieved 221 mg/L of total anthocyanins, of which 76% were non- acylated and 24% acylated. The vinifications after the sequential inoculation of the non-*Saccharomyces* yeasts *W. pratensis*, *L. thermotolerans* and the mixed L&T inocula did not achieve significant anthocyanin content compared to *S. cerevisiae*. The vinification performed initially by *M. pulcherrima* reached an anthocyanin content of 303 mg/L which was 37% significantly higher than that observed for *S. cerevisiae*. This total concentration was made up/composed of 78% non-acylated and 22% acylated anthocyanins. When sequential fermentation with *T. delbrueckii*, the total anthocyanins were 295 mg/L, which was 33% more than that observed with *S. cerevisiae*; of these, 76% were non-acylated and 24% acylated. In the case of fermentations with *Z. bailii*, the anthocyanin content was increased by 36% more than with *S. cerevisiae* and in vinifications in which *C. zeylanoides* was involved this increase was 34% higher. In both cases, the ratio of non-acylated and acylated was the same as that observed for *S. cerevisiae* and *T. delbrueckii*.

Table 2 shows average data (*n* = 2) of anthocyanins and stilbenes content of wine from sequential inoculation of two genotypes of *M. pulcherrima*, *T. delbrueckii*, and *L. thermotolerans* yeasts species. Statistical analysis showed in the petunidin-3-glucoside after *L. thermotolerans* genotype 57 was significantly lower than that assessed for genotype 54. No other significant differences were found among clones of the same species.

**Table 2:** Average anthocyans and stilbenes content (*n* = 2) in wines sequentially fermented with two clones of the yeast species *M.p*: *Metschnikowia pulcherrima*, *T.d*: *Torulaspora delbrueckii*, and *L.th*: *Lachancea thermotolerans.*

Compounds	*M.p.* 28	*M.p.* 29	*T.d.* 18	*T.d.* 19	*L.th.* 54	*L.th*. 57
**Anthocyanins (mg/L)**						
Non-acylated:						
Dp-3-glc	18.1 ± 2.9	18.5 ± 3.2	14.7 ± 0.8	17.1 ± 0.2	16.9 ± 3.1	15.2 ± 0.27
Cn-3-glc	1.22 ± 0.11	1.25 ± 0.08	1.12 ± 0.00	1.34 ± 0.08	1.44 ± 0.09	1.27 ± 0.03
Pt-3-glc	32.8 ± 2.6	33.8 ± 3.4	29.2 ± 0.4	29.6 ± 0.5	24.4 ± 3.6	22.8 ± 0.5
Pn-3-glc	7.82 ± 1.03	7.49 ± 0.84	8.71 ± 1.37	10.5 ± 0.5	11.9 ± 0.7b	8.66 ± 0.00a
Mv-3-glc	176 ± 8	175 ± 7	166 ± 0	167 ± 2	139 ± 9	135 ± 3
Acetylated:						
Dp-3-acglc	4.92 ± 0.08	5.05 ± 0.82	4.29 ± 0.19	4.29 ± 0.04	3.65 ± 0.06	3.57 ± 0.14
Cn-3-acglc	0.78 ± 0.02	0.78 ± 0.01	0.75 ± 0.01	0.76 ± 0.02	0.71 ± 0.01	0.69 ± 0.04
Pt-3-acglc	2.12 ± 0.06	2.16 ± 0.14	1.97 ± 0.04	1.85 ± 0.12	1.61 ± 0.12	1.63 ± 0.13
Pn-3-acglc	0.82 ± 0.01	0.86 ± 0.01	0.80 ± 0.02	0.84 ± 0.01	0.81 ± 0.01	0.81 ± 0.03
Mv-3-acglc	7.10 ± 0.19	7.13 ± 0.21	6.66 ± 0.11	6.8 ± 0.0	5.67 ± 0.34	5.68 ± 0.31
Coumaroylated:						
Dp-3-cmglc	5.44 ± 0.24	5.38 ± 0.69	6.37 ± 0.06	6.01 ± 0.29	4.69 ± 0.52	4.19 ± 0.20
Cn-3-cmglc	1.24 ± 0.00	1.24 ± 0.06	1.37 ± 0.02	1.33 ± 0.03	1.19 ± 0.06	1.06 ± 0.03
Pt-3-cmglc	4.88 ± 0.19	4.70 ± 0.50	5.77 ± 0.70	4.93 ± 0.09	4.08 ± 0.48	3.57 ± 0.05
Pn-3-cmglc	4.46 ± 0.14	4.33 ± 0.22	5.27 ± 0.22	5.02 ± 0.05	4.67 ± 0.30	3.65 ± 0.00
Mv-3-*cis*-cmglc	1.33 ± 0.19	1.18 ± 0.03	1.40 ± 0.01	1.47 ± 0.01	1.63 ± 0.05	1.42 ± 0.23
Mv-3-*trans*-cmglc	33.6 ± 1.5	32.0 ± 2.1	37.2 ± 0.4	36.2 ± 0.2	30.9 ± 2.1	27.1 ± 0.6
Caffeoylated:						
Mv-3-cfglc	1.55 ± 0.08	1.54 ± 0.21	1.49 ± 0.12	1.67 ± 0.04	1.58 ± 0.23	1.30 ± 0.11
**Stilbenes (mg/L)**						
*Trans* -piceid	0.32 ± 0.03	0.31 ± 0.06	0.31 ± 0.04	0.28 ± 0.02	0.33 ± 0.10	0.28 ± 0.03
*Cis*-piceid	0.75 ± 0.03	0.80 ± 0.00	0.83 ± 0.01	0.83 ± 0.00	0.71 ± 0.07	0.62 ± 0.03
*Trans*-resveratrol	0.12 ± 0.01	0.12 ± 0.02	0.19 ± 0.03	0.15 ± 0.02	0.09 ± 0.03	0.07 ± 0.02


*Dp, delphinidin; Cn, cyanidin; Pt, petunidin; Pn, peonidin; Mv, malvidin; glc, glucoside; acglc, acetylglucoside; cmglc, *trans*-*p*-coumaroylglucoside; cfglc, caffeoylglucoside. All parameters are listed with their standard deviation (*n* = 2). No letters mean no significant differences.*

Table 3 shows the detailed anthocyanin average content analyzed in each of the fermentation processes. The content of cyanidin-3 acetylglucoside and of malvidin-3-caffeoylglucoside were not significantly different between wines vinified with the different yeast species but several other significant differences were observed and described. *S. cerevisiae* fermentation was characterized by the lowest values of all the non-acylated anthocyanins and all the coumaroylated anthocyanins. The resulting wine of the sequential inoculation of *W. pratensis* had the lowest values of petunidin-3-glucoside, peonidin-3-glucoside, peonidin-3-acetylglucoside and malvidin-3-acetylglucoside. In the case of the mixed culture *L&T*, it was observed that the wine had the lowest values of petunidin-3-glucoside, petunidin-3.acetylglucoside, petunidin-3-coumaoylglucoside, peonidin-3-acetylglucoside and malvidin-3-acetylglucoside. When *L. thermotolerans* was early inoculated alone, the final wines achieved the lowest contents of delphinidin-3-acetylglucoside, petunidin-3-acetylglucoside and malvidin-3-acetylglucoside but also the highest of cyanidin-3-glucoside, peonidin-3-glucoside and malvidin-3-*trans*-coumaroylglucoside. The wine vinified with *M. pulcherrima* showed the highest levels of most of the non-acylated anthocyanins and the highest of malvidin-3-acetylglucoside. The wine made with *T. delbrueckii* had the highest cyanidin-3-glucoside, peonidin-3-glucoside, peonidin-3-coumaroylglucoside, malvidin-3-cus-coumaroylglucoside and malvidin-3-*trans*-coumaroylglucoside. *Z. bailii* and *C. zeylanoides* provided the wines with the highest content of most of the non-acylated anthocyanins, most of the acetylated ones and of delfinidin-3-coumaroylglucoside, cyanidin-3-coumaroylglucoside and petudin-3-coumaroylglucoside.

**Table 3:** Average anthocyans (mg/L) compounds in wines vinified with two strains of *Metschnikowia pulcherrima* (*M.p*), *Torulaspora delbrueckii* (*T.d*), *Lachancea thermotolerans* (*L.t*), *L.t*/*T.d* (*L&T*), *Zygosaccharomyces bailii* (*Z.b*), *Candida zeylanoides* (*C.z*), and *Saccharomyces cerevisiae* (*S.c*), with their standard deviations and results of their statistical assessment.

Anthocyanins (mg/L)	*M.p*	*T.d*	*L.th*	*L&T*	*Z.b*	*W.p*	*C.z*	*S.c*
**Non-acylated**								
Df-3-glc	18.3 ± 2.5b	15.9 ± 1.5ab	16.0 ± 2.0ab	11.7 ± 2.1ab	17.7 ± 10b	11.5 ± 1.7ab	17.4 ± 3.0b	8.80 ± 0.05a
Cn-3-glc	1.24 ± 0.08b	1.23 ± 0.14b	1.35 ± 0.11b	1.09 ± 0.08ab	1.25 ± 0.01b	0.99 ± 0.04ab	1.32 ± 0.10b	0.82 ± 0.13a
Pt-3-glc	33.3 ± 2.5b	29.4 ± 0.4ab	23.6 ± 2.3a	21.8 ± 3.2a	34.5 ± 0.6b	23.3 ± 1.7a	33.0 ± 4.6b	21.2 ± 0.1a
Pn-3-glc	7.65 ± 0.79ab	9.60 ± 1.33b	10.29 ± 1.93b	6.42 ± 1.13ab	5.99 ± 0.29ab	4.87 ± 0.54a	6.47 ± 0.79ab	3.38 ± 0.02a
Mv-3-glc	176 ± 6c	167 ± 1bc	137 ± 6ab	132 ± 13a	171 ± 1c	137 ± 5ab	168 ± 16c	135 ± 0a
**Acetylated**								
Df-3-acglc	4.98 ± 0.48abc	4.29 ± 0.11abc	3.61 ± 0.10a	3.74 ± 0.64ab	5.41 ± 0.09c	4.47 ± 0.50abc	5.13 ± 0.43bc	3.86 ± 0.08ab
Cn-3-acglc	0.78 ± 0.01	0.76 ± 0.01	0.70 ± 0.02	0.70 ± 0.04	0.80 ± 0.01	0.77 ± 0.02	0.80 ± 0.05	0.73 ± 0.06
Pt-3-acglc	2.14 ± 0.09bcd	1.91 ± 0.10abc	1.62 ± 0.10a	1.62 ± 0.16a	2.29 ± 0.01cd	1.79 ± 0.01ab	2.34 ± 0.23d	1.71 ± 0.03ab
Pn-3-acglc	0.84 ± 0.02abc	0.82 ± 0.03abc	0.81 ± 0.02ab	0.79 ± 0.03a	0.93 ± 0.03c	0.80 ± 0.01a	0.92 ± 0.02bc	0.76 ± 0.06a
Mv-3-acglc	7.12 ± 0.17b	6.74 ± 0.12ab	5.68 ± 0.26a	5.77 ± 0.53a	7.20 ± 0.12b	5.93 ± 0.19a	7.08 ± 0.49b	6.40 ± 0.06ab
**Coumaroylated**								
Df-3-cmglc	5.41 ± 0.42bcd	6.19 ± 0.27cd	4.44 ± 0.43ab	4.04 ± 0.57ab	6.96 ± 0.25d	4.77 ± 0.32abc	6.64 ± 0.64d	3.67 ± 0.31a
Cn-3-cmglc	1.24 ± 0.03bcd	1.35 ± 0.03cd	1.13 ± 0.08b	1.05 ± 0.10ab	1.39 ± 0.01d	1.16 ± 0.01bc	1.40 ± 0.01d	0.88 ± 0.03a
Pt-3-cmglc	4.79 ± 0.33ab	5.35 ± 0.64b	3.83 ± 0.40ab	3.53 ± 0.44a	5.38 ± 0.04b	3.99 ± 0.12ab	5.32 ± 0.42b	3.43 ± 0.29a
Pn-3-cmglc	4.40 ± 0.17bcd	5.15 ± 0.20c	4.16 ± 0.62abc	3.44 ± 0.44ab	4.54 ± 0.17bc	3.44 ± 0.01ab	4.48 ± 0.44bc	2.84 ± 0.12a
Mv-3-*cus*-cmglc	1.25 ± 0.14ab	1.44 ± 0.04ab	1.52 ± 0.18b	1.25 ± 0.13ab	1.28 ± 0.04ab	1.16 ± 0.00ab	1.25 ± 0.01ab	1.08 ± 0.06a
Mv-3-*trans*-cmglc	32.8 ± 1.8bcd	36.7 ± 0.6c	29.0 ± 2.5ab	26.4 ± 3.2ab	33.5 ± 0.8bc	26.3 ± 0.6ab	32.8 ± 2.2bc	25.0 ± 0.9a
**Caffeoylated**								
Mv-3-cfGlc	1.54 ± 0.13	1.58 ± 0.13	1.44 ± 0.22	1.40 ± 0.12	1.22 ± 0.01	1.48 ± 0.07	1.44 ± 0.20	1.55 ± 0.01


*Dp, delphinidin; Cn, cyanidin; Pt, petunidin; Pn, peonidin; Mv, malvidin; glc, glucoside; acglc, acetylglucoside; cmglc, *trans*-*p*-coumaroylglucoside; cfglc, caffeoylglucoside. All parameters are listed with their standard deviation (*n* = 2). Different letters mean significant differences between the samples (*p* ≤ 0.01). No letters mean no significant differences.*

**FIGURE 2:**
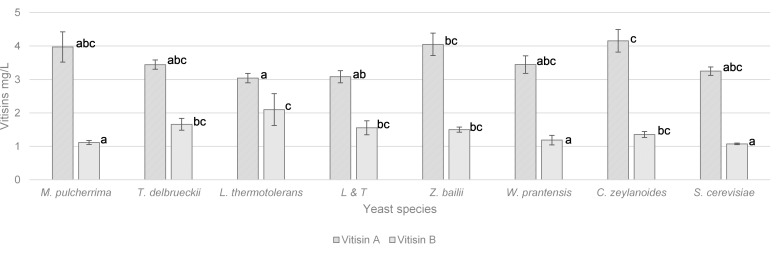
Vitisin A and B content (mg/L) and in wines vinified with different yeasts species. Error bars represent the standard deviation (*n* = 2). Different letters mean significant differences between the samples (*p* ≤ 0.01). No letters mean no significant differences.

The vitisin A and vitisin B contents are displayed in Figure 2. The highest content of vitisin A (around 4 mg/L) was found in wines vinified with *Z. bailii* and *C. zeylanoides* and the lowest (3 mg/L) in wine vinified with *L. thermotolerans*. With regard to vitisin B, *L. thermotolerans* was the vinification with the highest content (2 mg/L) while *M. pulcherrima*, *W. pratensis*, and *S. cerevisiae* had significantly lower concentrations of 50%.

**FIGURE 3:**
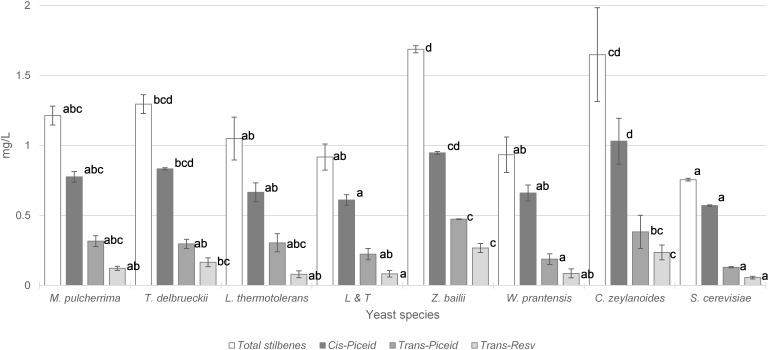
Total stilbene content (mg/L) with the most important groups of stilbenes in wine vinified with different yeasts species. Error bars represent the standard deviation (*n* = 2). Different letters mean significant differences between the samples (*p* ≤ 0.01). No letters mean no significant differences.

Concentrations of stilbenes in wines are shown in Figure 3. The vinifications performed only with *S. cerevisiae* provided the lowest values of total stilbenes, *cis*-piceid, *trans*-piceid and *trans*-resveratrol. The total stilbene content was significantly higher in vinifications performed initially by *T. delbrueckii*, *C. zeylanoides*, and *Z. bailii* that were respectively 71, 122, and 117% higher than *S. cerevisiae* vinification. The *cis*-piceid content of wines were 45, 66, and 80% higher in wines fermented initially with *T. delbrueckii*, *Z. bailii*, and *C. zeylanoides*, respectively. Regarding the *trans*-piceid content, it was double that the content in fermentation initiated with *Z. bailii* than in *S. cerevisiae* vinification and three times higher in fermentation initiated by *C. zeylanoides*. The *trans*-resveratrol concentration in wines inoculated with *T. delbrueckii* was three times higher than in winemaking using *S. cerevisiae* and 4.5 times higher when *Z. bailii* was inoculated.

## Discussion

The percentage of implantation of the non-*Saccharomyces* yeasts differed with the species yet their presence was progressively reduced since they were weeded in the initial must as it was described by Escribano-Viana et al. (2018). Just before *S. cerevisiae* inoculation, some species (*Z. bailii* and *W. pratensis*) were quickly lowered 3 days later. Every yeast species was able to establish in grape must under semi-industrial conditions. The yeasts that remained the longest time in tanks were *T. delbrueckii* and *L. thermotolerans*, widely participating in winemaking and probably in the wine’s features.

### Oenological Parameters of Wines

Comparing the data obtained in the non-*Saccharomyces*/*Saccharomyces* fermentations with those carried out with *S. cerevisiae* alone, the most significant changes were in the TPI value, glycerine, lactic and acetic acids, and ABV.

The glycerine content increased in wines fermented with *W. pratensis*, *L. thermotolerans/T. delbrueckii* mix, and mainly with *T. delbrueckii*. Glycerine is the second yeast metabolite in relevance that contributes to smoothness, sweetness and wine complexity. Different authors have previously informed that some non-*Saccharomyces* yeasts could synthetize high glycerine contents in winemaking, mainly in the studies involving *T. delbrueckii* (Belda et al., 2015b). On the other hand, an increase in the lactic acid content was observed in those fermentations where *L. thermotolerans* was present. The capacity of *L. thermotolerans* to produce lactic acid (Gobbi et al., 2013) caused great variations in pH and total acidity values between the wines vinified with this species. This characteristic of *L. thermotolerans* could be a tool to adjust the acidity level in wines (Benito et al., 2015). Regarding the volatile acetic acid concentrations, every non-*Saccharomyces* yeasts, except *L. thermotolerans*, tended to produce lower rates than *S. cerevisiae*, despite non-*Saccharomyces* yeasts has been described as producer of important acetic acid quantities. In addition, a reduction of the alcohol strength was noted in fermentations with the presence of *M. pulcherrima*, *T. delbrueckii*, and *L. thermotolerans.* The employment of non-*Saccharomyces* yeasts for the reduction of ethanol levels of wine has previously been indicated by other authors (Gonzalez et al., 2013; Contreras et al., 2015; Canonico et al., 2016).

Finally, the TPI value was increased with *C. zeylanoides*, *Z. bailii*, *T. delbrueckii* and particularly with *M. pulcherrima*. Belda et al. (2016) had previously indicated the potential of *Metschnikowia pulcherrima*, used on a semi-industrial scale combined with *S. cerevisiae*, to improve color properties in red wine due to its pectinolytic activities.

### Anthocyanin and Stilbenes Content After Alcoholic Fermentation

The results from the current work have confirmed that the wine’s phenolic composition, in particular anthocyanins and stilbenes, can be considerably modulated with the selection of a specific fermentation starter.

With respect to the monomeric anthocyanin composition, the results have shown that *M. pulcherrima*, *Z. bailii*, *C. zeylanoides*, and *T. delbrueckii* achieved the greatest increase of total anthocyanins when compared to *S. cerevisiae* and the other non-*Saccharomyces* yeasts. The importance of selecting the yeast starter in terms of wine color has been reported in previous works (Suárez-Lepe and Morata, 2012; Belda et al., 2015a, 2016). In this respect, *M. pulcherrima* has been proposed as a positive yeast for enhancing wine color. These authors also confirmed that *M. pulcherrima* displays polygalacturonase activity, which could explain the greater release of phenolic compounds during the AF. Sorrentino et al. (2012) also found that the combined inoculation of *M. fructicula* and a commercial *S. cerevisiae* yeast led to important improvements of wine anthocyanin content when compared with the commercial yeast alone.

*Torulaspora delbrueckii* has also been reported to increase anthocyanin content during AF. Recently, Chen et al. (2018) observed an increase in total anthocyanins for sequential fermentation of *T. delbrueckii*/*S. cerevisiae* compared with fermentation conducted by *S. cerevisiae* alone. On another note, *T. delbrueckii* has been also shown to modulate other phenolic compounds in wine. In this respect, Ngqumba et al. (2017) observed that the influence of *T. delbrueckii* on the phenolic composition of wine (i.e., flavonols and phenolic acids) of cv. Chenin blanc depended on the specific strain. This observation could be of great interest for selecting appropriate non-*Saccharomyces* strains for red wine production. Moreover, Carew et al. (2013) showed a high degree of tannin polymerization in wines made with *T. delbrueckii*/*S. cerevisiae*.

With respect to other yeast species, *S. pombe*, both alone or in combination with *L. thermotolerans* (Benito et al., 2015) improved the color of red wines by increasing several anthocyanins and vitisins with respect to *S. cerevisiae* alone. Moreover, these authors found that combined fermentation between *S. pombe* and *L. thermotolerans* or *S. cerevisiae* and *L. thermotolerans* showed higher concentrations in several anthocyanins than when *S. pombe* or *S. cerevisiae* fermented alone. The authors suggested that the latter result could be explained by a lower anthocyanin absorption by *L. thermotolerans* strain.

Benito et al. (2015) also observed that sequential fermentation with *P. guilliermondii* and *S. cerevisiae* both with high hydroxycinnamate decarboxylase (HCDC) activity promoted the formation of vinylphenolic pyranoanthocyanins, which are long-term stable pigments, without unwanted organoleptic variations. Moreover, this strategy allowed them to reduce the presence of *p*-coumaric acid (the precursor of 4-ethyphenol).

Regarding the influence of yeasts in stilbene composition, *C. zeylanoides*, *Z. bailii* and *T. delbrueckii* achieved the best results in terms of stilbene composition, especially when compared with *S. cerevisiae.* There is little information about the impact of wine microorganisms on wine stilbene composition. Since stilbenes are mainly located in grape skins, yeasts with enhanced enzymatic activities could presumably increase stilbene extraction from grape to wine. In this respect, treatment with pectinase before pressing was shown to increase stilbene content in Muscadine grape juice (Leblanc et al., 2008). In contrast, Lima et al. (2015) did not observe significant differences between doses of pectinase with regard to grape juice resveratrol content. Moreover, Gaensly et al. (2015) showed that differences in β-glucosidase activity could result in differences in wine resveratrol content. These authors observed that yeasts with β-glucosidase activity favored the hydrolysis of *trans*-piceid into *trans-*resveratrol without modifying the wine sensorial properties.

Overall, our results suggested that yeast selection has a great impact on quality, in particular on the anthocyanin and stilbene composition of wine. In this sense, the use of *M. pulcherrima, T. delbrueckii, Z. bailii*, and *C. zeylanoides* as fermentation starters could be of great interest in order to achieve wines with better color and health properties. These four yeast species provided an increase in TPI without increasing some negative organoleptic properties as acetic acid content. With the exception of *T. delbrueckii*, the other three species disappeared from the environment when *S. cerevisiae* was added, and therefore, the end of AF is under the control of the latter species, thereby avoiding the possible negative effects on other wine parameters. In the case of *T. delbrueckii*, it also produced a very significant increase in the concentration of glycerine, a compound that also improved the organoleptic perception of red wines.

## Author Contributions

All authors listed have made a substantial, direct and intellectual contribution to the work, and approved it for publication.

## Conflict of Interest Statement

The authors declare that the research was conducted in the absence of any commercial or financial relationships that could be construed as a potential conflict of interest.
